# Effects of a silver nanoparticle and fluoride solution on enamel remineralization: an *in situ* study

**DOI:** 10.1590/0103-644020267220

**Published:** 2026-10-19

**Authors:** Flaviana Alves Dias, Jaqueline Costa Favaro, Francielle Delgado Agostini, Thais Fernandes Poleti, Ricardo Danil Guiraldo, Murilo Baenla Lopes, Sandrine Bittencourt Berger

**Affiliations:** 1 Department of Restorative Dentistry, University of North Parana, Londrina, PR, Brazil; 2 Department of Restorative Dentistry, University Anhanguera/Uniderp, Campo Grande, MS, Brazil; 3 University of Connecticut - UConn School of Dental Medicine - Estados Unidos

**Keywords:** Calcium hydroxide, glass, bioactive, copper, endodontics

## Abstract

This *in situ* study evaluated the remineralizing effect of a silver nanoparticle and fluoride solution (NanoF) compared with silver diamine fluoride (SDF) on dental enamel with initial caries lesions. This blind, randomized, crossover *in situ* study was conducted in two experimental phases (10 days each), separated by a 10-day washout period. Eighteen volunteers (n = 18) used palatal devices containing six bovine enamel blocks: one sound enamel (SE), one initial caries lesion (IC), two treated with NanoF, and two treated with SDF. Initial caries lesions were formed *in situ* before treatment, and baseline surface microhardness (Mi) was obtained. Participants then applied a 20% sucrose solution to the samples eight times daily for eight days to induce demineralization, followed by a second microhardness assessment (Ml). After treatment, final surface microhardness (Mf) was measured, and the percentage of surface microhardness recovery (%SMR) was calculated. Cross-sectional microhardness (CSMH) was assessed after longitudinal sectioning of the samples, and mineral volume percentage (%MV) was calculated at depths of 20-200 μm. Data were analyzed using Kruskal-Wallis followed by Dwass-Steel-Critchlow-Fligner tests (α = 0.05). NanoF showed %SMR values statistically similar to SDF and SE, but significantly higher than IC (p = 0.028). For CSMH, NanoF and SDF did not differ, whereas SE presented higher values and IC the lowest values (p < 0.001), regardless of depth. NanoF demonstrated comparable *in situ* remineralization potential to SDF in initial enamel caries lesions.

## Resumo

As limitações estéticas do diamino fluoreto de prata estimularam o desenvolvimento de alternativas remineralizadoras à base de nanopartículas de prata. Este estudo *in situ* avaliou o efeito remineralizador de uma solução contendo nanopartículas de prata e flúor (NanoF), em comparação ao diamino fluoreto de prata (DFP), no esmalte dental com lesão inicial de cárie. Trata-se de um estudo in situ, cego, randomizado e cruzado, conduzido em duas fases experimentais (10 dias cada), separadas por um período de washout de 10 dias. Dezoito voluntários (n = 18) utilizaram dispositivos palatais contendo seis blocos de esmalte bovino: um de esmalte sadio (ES), um com lesão inicial de cárie (LC), dois tratados com NanoF e dois tratados com DFP. As lesões iniciais de cárie foram formadas in situ antes do tratamento, e a microdureza superficial inicial (Mi) foi obtida. Em seguida, os participantes aplicaram uma solução de sacarose a 20% nas amostras oito vezes ao dia, durante oito dias, para induzir a desmineralização, sendo realizada nova avaliação da microdureza (Ml). Após os tratamentos, a microdureza superficial final (Mf) foi mensurada, e calculou-se a porcentagem de recuperação da microdureza superficial (%MS). A microdureza transversal foi avaliada após o seccionamento longitudinal das amostras, e o volume mineral percentual (%VM) foi calculado em profundidades de 20 a 200 μm. Os dados foram analisados pelos testes de Kruskal-Wallis e Dwass-Steel-Critchlow-Fligner (α = 0,05). O NanoF apresentou valores de %MS estatisticamente semelhantes aos do DFP e do ES, e significativamente superiores aos do grupo LC (p = 0,028). Para a %VM, NanoF e DFP não diferiram entre si, enquanto o ES apresentou valores superiores e o LC os menores valores (p < 0,001), independentemente da profundidade. O NanoF promoveu a remineralização de lesões iniciais de cárie no esmalte, com desempenho comparável ao DFP, indicando seu potencial como alternativa para o manejo de lesões iniciais do esmalte.

**Figure:**
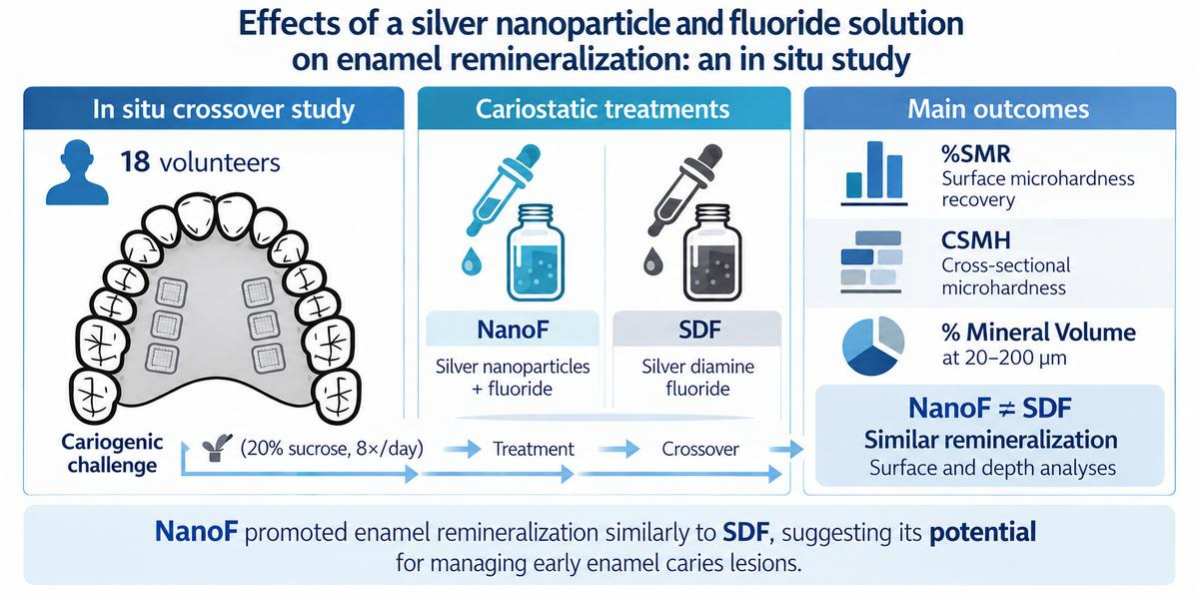

## Introduction

Dental caries is initially characterized by localized chemical dissolution of hard dental tissues, caused by the metabolic processes of biofilms located on the dental surface ^1^ ^,^ ^2^. Protocols for arresting and preventing dental caries have been suggested, which use compounds that promote demineralization balance and antimicrobial strategies, differing from traditional restorative methods. ^1^ An effective agent in the prevention and control of dental caries is silver diamine fluoride (SDF), characterized as cariostatic and preventive ^3^. In the 1960s, SDF was approved for therapeutic use in Japan ^4^. It has been used to arrest caries, treat established lesions in primary teeth, and prevent caries in the pits and fissures of erupting permanent molars ^3^, as well as being used to prevent root caries ^5^ ^,^ ^6^. The mechanism of action of SDF is well established. It involves the release of silver (Ag⁺) and fluoride (F⁻) ions, which interact with hydroxyapatite to form calcium fluoride (CaF₂)-like deposits and fluorohydroxyapatite, contributing to remineralization ^2^ ^,^ ^7^. Additionally, silver ions react with phosphate groups to form silver phosphate (Ag₃PO₄) and other silver-based precipitates. These precipitates are associated with arresting the progression of carious lesions and staining dental tissues. ^2^ ^,^ ^8^. This staining is mainly attributed to the reduction of Ag⁺ to metallic silver (Ag⁰) and the formation of light-sensitive compounds during oxidation-reduction reactions ^3^ ^,^ ^8^, which may result in aesthetic dissatisfaction and consequently limit its use in anterior teeth ^3^ ^,^ ^9^.

In this way, silver nanoparticle (AgNP)-based solutions have been studied and have been shown to be effective in preventing and arresting dental caries without staining demineralized tooth enamel. ^5^ ^,^ ^9^ ^,^ ^10^ ^,^ ^11^ ^,^ ^12^. AgNPs exhibit physicochemical behavior different from that of SDF. These particles consist predominantly of metallic silver (Ag⁰) stabilized at the nanoscale, acting as reservoirs for the controlled release of Ag⁺ rather than promoting immediate ionic precipitation ^13^. This controlled ion release reduces the formation of chromogenic silver compounds, explaining the absence of visible staining in AgNP-based formulations ^9^ ^,^ ^14^. Furthermore, due to their high surface area and small size, AgNPs can interact more effectively with bacterial biofilms, penetrating cell membranes, disrupting metabolic processes, and inhibiting DNA replication ^5^ ^,^ ^12^ ^,^ ^13^.

When combined with fluoride, AgNPs provide a synergistic effect that involves both antimicrobial activity and remineralization. Fluoride ions promote the formation of fluorapatite and reduce enamel dissolution, while AgNPs provide antibacterial properties ^2^ ^,^ ^13^ ^,^ ^15^. Unlike SDF, which forms surface precipitates, AgNP-based systems may promote more controlled and homogeneous interaction at the enamel interface, potentially resulting in a distinct remineralization pattern without compromising esthetics ^9^ ^,^ ^13^. In vitro studies have demonstrated the antibacterial properties, capacity to enhance remineralization, and minimal cytotoxicity of nano silver fluoride as a promising anticaries agent ^9^ ^,^ ^12^ ^,^ ^15^. Clinical studies indicate that nano silver fluoride may improve the remineralization of initial enamel lesions and arrest the advancement of dentin caries ^10^ ^,^ ^16^. Furthermore, Favaro et al. ^9^ tested a nano silver fluoride solution that exhibits long-term stability, significant antimicrobial activity against cariogenic microorganisms, and the ability to promote enamel and dentin remineralization ^5^, with performance comparable to SDF, yet without causing tooth discoloration.

Although these laboratory findings are encouraging, there is a lack of studies evaluating this association under clinically relevant conditions, particularly in *in situ* protocols that more accurately replicate the oral environment. Thus, assessment of the remineralizing impact of silver nanoparticles combined with fluoride on enamel with early carious lesions is needed to verify its effectiveness and safety in more realistic conditions. Based on the aforementioned, the present study aimed to test the hypothesis that an experimental solution containing AgNPs (NanoF) can promote *in situ* enamel remineralization in the presence of an initial lesion, both superficially and deeply, with a similar effect to that of SDF.

## Methods

The current study received approval from the Human Ethical Committee (Protocol: 01631918.0.0000), and all participants provided informed consent by reading and signing consent forms before the study began. This double-blind, crossover *in situ* study was conducted in two phases, each lasting 8 days, separated by a 10-day washout period.

### Sample size calculation

The sample calculation was based on the results of Scarpelli et al. ^11^. The mean enamel remineralization values and standard deviations for the SDF and NanoF solutions were used in the analysis. A mean enamel remineralization of 28.55% (±11.75) for SDF and 14.63% (±13.38) for NanoF was considered, with an α error of 5% and a statistical power of 80%. The calculation indicated that at least 13 participants would be required to detect significant differences between treatments. Thus, a total of 18 participants were recruited to mitigate the potential impact of dropouts or non-adherence to the protocol.

### Preparation of the bovine enamel specimens

Eighty bovine incisors, free of caries and enamel defects, were cleaned with periodontal curettes and subjected to prophylaxis using pumice and a Robinson brush to ensure complete debris removal. The teeth were then stored in 0.5% chloramine-T for 7 days and subsequently kept in distilled water at 4 °C until the beginning of the study. Enamel blocks measuring 4 mm × 4 mm × 3 mm were prepared from these teeth. The enamel surfaces were flattened using silicon carbide papers of grit #600, #1200, and #2000, followed by polishing with felt discs (Arotec S/A Ind. e Comércio) and diamond pastes of 1 µm and ¼ µm under low rotation using a polishing machine (Arotec S/A Ind. e Comércio). After each polishing step, the blocks were rinsed in an ultrasonic bath with distilled water (Unique Ind. e Co. Produtos Eletrônicos Ltda, São Paulo, SP, Brazil) for 10 minutes to remove any residues. The samples were then sterilized in an autoclave (Odontoplas, São Paulo, SP, Brazil) at 121 °C for 20 minutes ^17^.

### Division of specimens into experimental groups

Initially, the enamel microhardness of all samples was measured using a Shimadzu microhardness tester (HMV-G 21S, Shimadzu Corporation, Japan). A Knoop-type pyramidal diamond indenter with a static load of 25 grams was used for 10 seconds ^18^. Three indentations were made in each sample, with a distance of 100 μm between them. The average of the three indentations in each sample represents the initial microhardness (M_*i*_ ). The M_*i*_ values were used to select the samples. The overall microhardness average was calculated, and specimens with values below and above 10% of the average were discarded from the study. Thus, 216 blocks that met the microhardness inclusion criteria were randomly divided (using the Excel =RANDOM function) into four experimental groups, as described in Table 1.

Additionally, Table 1 describes the composition of the solutions. NanoF is an experimental solution that has been tested previously. Favaro et al. ^9^ characterized the physicochemical properties, antimicrobial activity, stability, and remineralizing potential of this formulation.

After distribution and randomization of the specimens, the groups were statistically analyzed for normality and homoscedasticity. The data were tested using ANOVA, which revealed that the groups were statistically equal before treatment (p = 0.575).

**Table 1:** Experimental groups according to treatments

Group	Enamel	Treatment	Composition
SE	Sound	Without treatment	-----
IC	Initial Caries Lesion	Without treatment	------
^§^SDF	Initial Caries Lesion	Silver Diamine Fluoride	30% Silver Nitrate, Ammonium Hydroxide, Hydrochloric Acid and Deionized Water
NanoF	Initial Caries Lesion	Fluoridated solution containing silver nanoparticles	0.04% colloidal silver nanoparticles with size from 20-100 nm (average size 30nm), ethylene glycol, polyvinylpyrrolidone, water

^§^SDF, Cariestop® (Biodinâmica, Ibiporã, Paraná, Brazil).

### *In situ* phase

This is an *in situ*, randomized, crossover study with two experimental periods (Figure 1). Each participant was exposed to only one agent per phase (NanoF or SDF), and a 10-day washout period was implemented before crossover to the second agent, ensuring no simultaneous presence of different treatments in the oral cavity. 

Eighteen participants were selected for the study based on the following inclusion criteria: age between 18 and 30 years; normal salivary flow rate; good general and oral health; and the ability to comply with the experimental protocol. The participants used an intraoral palatal device for two experimental periods, each corresponding to treatment with one of two proposed cariostatic substances: NanoF and SDF. Participants were excluded if they had fewer than 22 natural teeth, caries lesions, gingivitis, periodontal disease or other oral pathology; if they had used antibiotics or any other medication that modifies salivary flow in the two months before the study; if they had any systemic disease; if they were pregnant or breastfeeding; if they were undergoing orthodontic treatment with fixed or removable appliances; if they were using fluoride mouthwashes routinely; or if they had hyposalivation ^19^.

Each device had six niches located bilaterally in the molar region, with three on each side, into which the bovine tooth enamel samples were placed. Plastic grids were placed over the samples, leaving a 1.0 mm gap between the grid and the tooth surface to allow bacterial biofilm to accumulate (Figure 1). Each experimental period consisted of two phases. The first phase involved an eight-day period, during which the samples were subjected to a cariogenic challenge to induce initial caries. The second phase lasted ten days, beginning with the application of one cariostatic agent to the enamel specimens. Prior to initiating the subsequent experimental period and treatment sequence, a 10-day washout interval was implemented to minimize residual effects and standardize baseline conditions for all participants ^19^. The participant was considered the experimental unit (n = 18), as multiple enamel blocks were allocated within the same palatal device.

### Cariogenic challenge

Participants used palatal devices containing five enamel samples (SE group samples were excluded from this phase). They dropped 20% sucrose solution onto the surface of the plastic grid of each sample eight times a day for eight days. Drops were delivered at the following times: 8 am, 09:30 am, 11 am, 2 pm, 3:30 pm, 5 pm, 7 pm, and 9 pm ^20^. Participants were instructed to place each drop of solution onto the surface of the plastic grid without touching it, and to keep the device in a container with moistened cotton for five minutes before placing it in their mouth. 

All participants used the same fluoride toothpaste (Oral-B® Pro-Saúde Advanced, manufactured by Procter & Gamble in San Andrés, Mexico) three times a day. They brushed the buccal surfaces of all teeth and the lingual surfaces of the lower teeth with the device positioned in the arch. They then removed the device to brush the remaining surfaces. Participants were instructed to reposition the device in their mouths for the final rinsing. During the first phase, enamel blocks were subjected to a 20% sucrose challenge eight times daily for 8 days to induce subsurface caries-like lesions ^21^. After this period, the sucrose challenges were discontinued, and the experimental treatments were applied. After Phase 1, bovine tooth enamel samples were taken from the devices to assess enamel demineralization using surface microhardness readings (M_*l*_ ).

**Figure 1:**
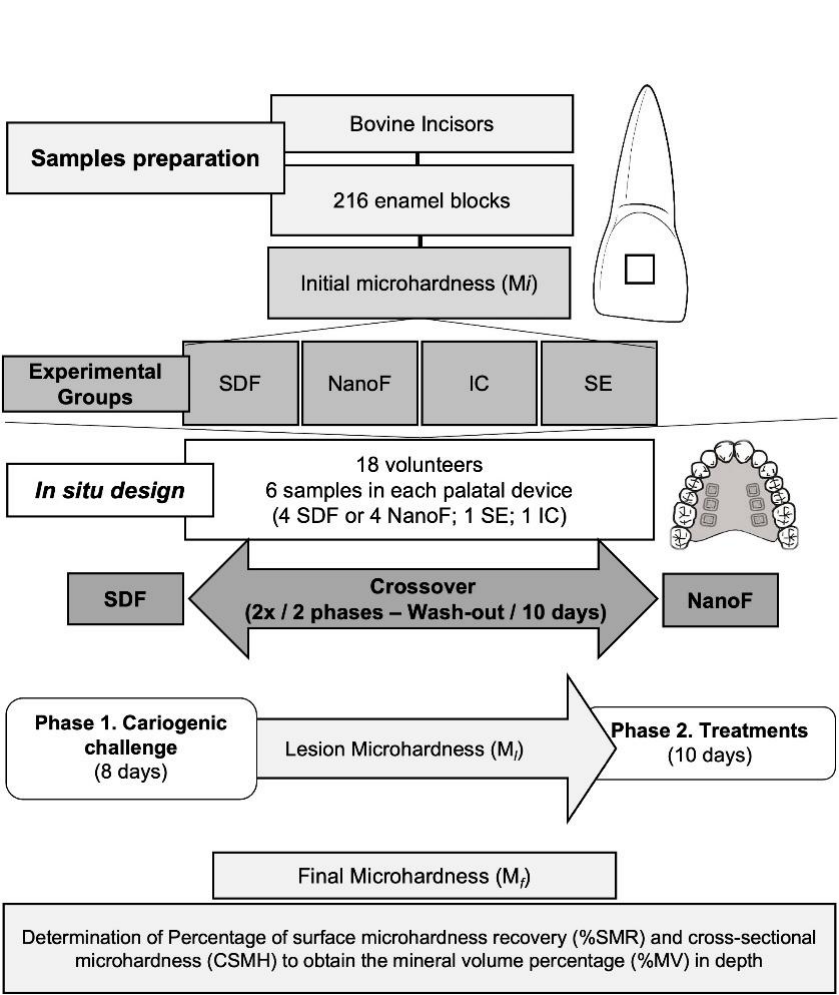
Experimental design diagram. SE - sound enamel; IC - initial caries lesion; SDF - silver diamine fluoride; NanoF - fluoridated solution containing silver nanoparticles.

### Treatments

After prophylaxis using a Robison brush, water, and a pumice stone, the samples from the SDF and NanoF experimental treatment groups were washed and dried before receiving a three-minute application of solutions using a disposable applicator on the enamel surface. During the first experimental period, one half of the participants used the device with SDF samples for 10 days, while the other half used it with NanoF samples. After this time, the devices were collected and the samples removed from the plates for analysis of the final surface microhardness (M_*f*_ ). After a 10-day interval (washout), new samples were placed in the plates to begin the second experimental period. Participants who had received SDF-treated specimens in the first period received NanoF-treated specimens in the second period, and vice versa (crossover). One sample from the SE group (negative control) and one from the IC group (positive control) were placed in the devices alongside four samples treated with SDF or NanoF in the second phase of the experimental period.

### Determination of percentage of surface hardness recovery (%SMR)

The initial surface microhardness values (M_*i*_ ), the values after the cariogenic challenge (M_*l*_ ), and the final values (M_*f*_ ) were used to calculate the percentage of surface enamel remineralization (%SMR) using the following formula:

$$\%SMR=\frac{Mf-Ml}{Mi-Ml}\times 100$$

### Cross-sectional microhardness (CSMH)

The bovine tooth enamel samples were longitudinally sectioned through the center of the previously exposed area, producing two matching halves. One half was embedded in acrylic resin, and its internal surface was progressively polished. Using a microhardness tester (HMV-G 21S, Shimadzu Corporation, Japan), three rows of ten Knoop indentations were made in the central region of the specimen under a 25 g load for 10 seconds, positioned at 20, 40, 60, 80, 100, 120, 140, 160, 180, and 200 μm from the outer enamel surface. After determining the CSMH, the data were analyzed to calculate the mineral volume percentage (%MV) at different depths, using the following formula ^22^:

$$\%MV=4.3\times \sqrt{KHN}+11.3$$

### Blinding

The operator responsible for treatment application was blinded to group allocation, as the materials were provided in a ready-to-use format and applied using a microbrush. However, blinding of the examiner responsible for the microhardness assessment was not feasible due to the characteristic staining produced by SDF on the enamel surface, which made group identification unavoidable. A blinded investigator conducted the statistical analysis.

### Statistical analysis

The participants were considered the experimental units, and the mean value of the enamel blocks within each participant was used for statistical analysis in order to mitigate the effects of clustering. All analyses were performed using Jamovi software (version 2.5.3.0, The Jamovi Project, Sydney, Australia) adopting a significance level of p < 0.05. The data were analyzed for normality and homoscedasticity using the Shapiro-Wilk and Levene tests, respectively. As the %SMR and %VM data were not normally distributed, they were analyzed using the Kruskal-Wallis test, followed by the Dwass-Steel-Critchlow-Fligner (DSCF) test.

## Results

Table 2 presents the comparisons of the percentage of %SMR in different groups after the application of the cariostatic solutions.

**Table 2:** Median, minimum and maximum of %SMR according to the experimental groups.

Experimental groups	%SMR Median/Mimimum/Maximum	DSCF test
SE	97.7 / 53.8 / 134.2	B
IC	42.0 / -106.6 / 115.2	A
SDF	63.7 / 19.8 / 98.0	BC
NanoF	68.3 / 37/2 / 119.2	C

Different letters indicate statistically significant differences, p<0.05. SE - sound enamel; IC - initial caries lesion; SDF - Silver Diamine Fluoride; NanoF **-** Fluoridated solution containing silver nanoparticles at 0.016%. DSCF - Dwass-Steel-Critchlow-Fligner.

The results showed that the IC group exhibited significantly lower values than all other groups (p < 0.05). The SDF group did not differ significantly from either the SE group (p = 0.293) or the NanoF group (p = 0.792), and both groups demonstrated higher values than the IC group.

Figure 2 presents the CSMH data. The NanoF group did not differ significantly from the SDF group (p = 0.603) but showed significantly higher values than the IC group (p < 0.001) and lower values than the SE group (p = 0.003). The IC group exhibited the lowest values, differing from all other groups (p < 0.001). The SE group showed significantly higher values compared with the SDF group (p < 0.001). When comparing depths, regardless of treatment group, a significant difference was observed only between 20 µm and 200 µm (p = 0.010).

**Figure 2:**
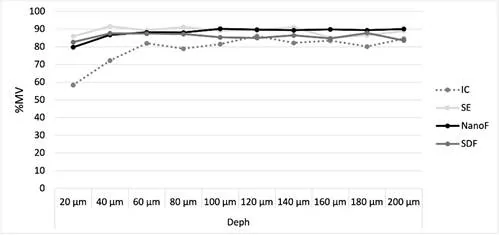
Graphical representation of CSMH values according to depth. SE - sound enamel; IC - initial caries lesion; SDF - silver diamine fluoride; NanoF - fluoridated solution containing silver nanoparticles.

## Discussion

The current study compared the remineralizing action of two caries-inhibiting agents containing fluoride and silver; NanoF (an experimental agent containing silver nanoparticles) and SDF, on enamel with initial caries lesions with initial caries lesion, using an *in situ* model. Both the surface and depth of the treated groups showed a remineralizing effect on the initial caries lesion, with values higher than those of untreated enamel. There was no significant difference between the two agents (SDF or NanoF). Therefore, the hypothesis tested in the study was accepted.

AgNPs can penetrate carious lesions and bind to hydroxyapatite crystals. Additionally, silver ions released by these nanoparticles can produce insoluble silver chloride in hard dental tissues ^23^. AgNPs and precipitated insoluble silver chloride increase the mineral density of these tissues ^13^. When AgNPs associate with fluoride ions, they can bind to calcium and phosphate ions to form fluorapatite and fluorhydroxyapatite, which are more acid-resistant than hydroxyapatite ^13^. The present study observed an increase in surface microhardness values after the application of the cariostatic agents SDF and NanoF. These values were statistically superior to those of the untreated carious control group (SE), which suggests remineralization of the previously demineralized enamel surface. The two solutions produced similar results. These findings differ from those of Scarpelli et al.^11^, who demonstrated an increase in microhardness values with the AgNPs solution that was lower than that of the SDF group. This discrepancy may be explained by the addition of fluoride and its stabilization in the AgNPs solution formulation in the present study, which could have increased enamel mineral density during remineralization.

Surface microhardness is widely used as an endpoint to assess mineral gain; however, it does not fully represent caries arrest. Caries arrest is a multifactorial process involving not only mineral recovery but also biofilm modulation, reduction of microbial activity, and stabilization of the lesion ^1^ ^,^ ^2^. Therefore, although the increase in microhardness observed in this study suggests remineralization, it should not be interpreted as definitive evidence of lesion arrest. In addition, the present study did not include direct assessment of biofilm composition or microbial activity. This represents an important limitation, as dental caries is a biofilm-mediated disease ^1^ ^,^ ^2^. Although previous studies have demonstrated the antimicrobial potential of silver nanoparticles against cariogenic microorganisms^9^ ^,^ ^10^ ^,^ ^24^, the absence of microbiological evaluation in the current model limits conclusions regarding the effect of NanoF on biofilm modulation.

Silver diamine fluoride (SDF) reacts with hydroxyapatite, the mineral component of dental enamel, resulting in the formation of calcium fluoride (CaF₂) and silver-containing compounds, including silver phosphate (Ag₃PO₄), which play an important role in caries prevention and lesion hardening. Calcium fluoride acts as a fluoride reservoir, releasing fluoride ions that, in the presence of calcium and phosphate, promote the formation of fluorapatite or fluorhydroxyapatite. Evidence indicates that SDF significantly increases the surface microhardness of demineralized enamel. The formation of fluorhydroxyapatite crystals that firmly adhere to the carious lesion contributes to enamel remineralization and caries arrest. These mechanisms are consistent with previous findings and corroborate the results of the present study, in which SDF significantly remineralized enamel with initial caries lesions ^7^ ^,^ ^25^. Overall, silver diamine fluoride (SDF) is an effective agent for caries arrest and prevention, exhibiting well-documented remineralizing and antimicrobial properties ^2^ ^,^ ^25^ ^,^ ^26^ ^,^ ^27^.

Although silver diamine fluoride (SDF) is effective, its application is related to dark staining of demineralized dental tissues, which may cause patient dissatisfaction. Silver nanoparticle (AgNP)-containing cariostatic agents have been suggested as a different approach in this situation. The ability of AgNP-based agents to avoid surface staining at the application site is a well-known benefit. ^9^ ^,^ ^10^ ^,^ ^11^ ^,^ ^28^ These substances aim to maintain the cariostatic and antimicrobial qualities of SDF while reducing its aesthetic disadvantages. AgNPs have been shown to stop the caries process effectively and completely inhibit microbial activity without causing discoloration from silver precipitation ^9^ ^,^ ^10^ ^,^ ^11^ ^,^ ^12^ ^,^ ^28^.

Clinical results corroborate our findings, showing similar effectiveness of AgNPs (NanoF group) and SDF fluoride-based solutions in halting the carious process ^29^. As with clinical studies, *in situ* studies can be influenced by the complexity and dynamic balance of demineralization and remineralization in the presence of oral fluids and complex biofilms. This resembles the remineralization results between solutions. AgNPs are considered to have an antibacterial mechanism involving the release of silver ions from the particles onto the bacterial cell membrane, thereby altering its structure and permeability. This allows AgNPs and silver ions to penetrate the cell interior, causing direct and indirect protein and lipid peroxidation. This, in turn, modifies organelles, cell transport, DNA replication, and protein activity within the bacterial cell. In the study by Targino *et al*. (2014) ^12^, the AgNP fluoride-based solution was effective against *S. mutans* without exhibiting cytotoxic effects on human erythrocytes. The same agent produced positive results in preventing the formation of cariogenic biofilms ^9^ ^,^ ^10^ ^,^ ^24^ and halting the caries process ^10^.

These *in vitro* findings are similar to previous studies that tested solutions based on silver nanoparticles associated with fluoride for remineralizing dental enamel with initial caries lesions. Previous randomized clinical trial studies verified the use of a fluoridated solution based on silver nanoparticles for preventing dental enamel caries ^10^ ^,^ ^16^. The authors stated that this effect could be explained by the synergistic action of the formulation's components. Nozari et al. ^15^ and Favaro et al. ^9^ observed the same synergy in an *in vitro* study with human deciduous teeth and human teeth, respectively. The results showed that the highest remineralization values were obtained through the combination of silver nanoparticles and fluoride. Therefore, the action of silver nanoparticles can be enhanced by combining them with fluoride ^14^, with both being responsible for the remineralization of dental enamel ^15^.

The present study also assessed the stronger effects of SDF and NanoF. As shown in Figure 2, both promoted remineralization, in contrast to the group with initial caries lesions (without treatment), indicating sound dental enamel. These data are supported by the literature, which shows that both SDF and NanoF can remineralize the deeper substrate when internal microhardness is measured ^2^ ^,^ ^9^. However, as observed in the %MV data, the demineralization promoted by the protocol for forming initial caries lesions was more superficial than deep. Additionally, the demineralization promoted by the protocol for creating initial caries lesions was more superficial than deep, as indicated by the mineral volume values shown in Figure 2.

Although this is an *in situ* study, some limitations should be considered. *In situ* studies using intraoral appliances for remineralization present variability in salivary exposure and oral dynamics among volunteers, which introduces heterogeneity in the outcomes, even when rigorous criteria are followed for the inclusion and exclusion of participants. For example, factors such as the nature and flow rate of saliva, diet, oral hygiene, and individual behavior (e.g., frequency of appliance removal, eating, and drinking) are difficult to standardize and control across participants ^30^. In addition, *in situ* models do not fully reproduce the heterogeneity of clinical caries lesions or include clinically relevant patient outcomes, which should be considered when extrapolating these findings to clinical practice ^16^ ^,^ ^25^. Furthermore, the initial condition of enamel lesions, including the depth and extent of demineralization and lesion type (e.g., surface-softened versus subsurface lesions), can influence remineralization rates ^31^, and residual effects between crossover phases cannot be completely excluded, despite the washout period adopted ^19^.

## Conclusion

NanoF promoted the remineralization of initial enamel caries lesions *in situ*, showing results comparable to SDF. These results suggest that NanoF can be an effective, non-staining remineralizing treatment. Further clinical studies are needed to confirm effectiveness.

## Data Availability

The research data are available upon request.
